# Circulating, Extracellular Vesicle-Associated Tissue Factor in Cancer Patients with and without Venous Thromboembolism

**DOI:** 10.3390/biom15010083

**Published:** 2025-01-08

**Authors:** Valentina Lami, Dario Nieri, Marta Pagnini, Mario Gattini, Claudia Donati, Mariella De Santis, Alessandro Cipriano, Erica Bazzan, Andrea Sbrana, Alessandro Celi, Tommaso Neri

**Affiliations:** 1 UO Medicina d’Urgenza e Pronto Soccorso, Azienda Ospedaliero-Universitaria Pisana, 56124 Pisa, Italy; valentina.lami@gmail.com (V.L.);; 2 UO Pneumologia, Azienda Ospedaliero-Universitaria Pisana, 56124 Pisa, Italy; darionieri@hotmail.it (D.N.); alessandro.celi@unipi.it (A.C.); 3 Dipartimento di Patologia Chirurgica, Medica, Molecolare e dell’Area Critica, University of Pisa, 56126 Pisa, Italy; marta.pagnini@med.unipi.it (M.P.); mariogattini96@gmail.com (M.G.); claudiadonati24@gmail.com (C.D.); 4 Centro Dipartimentale di Biologia Cellulare Cardiorespiratoria, University of Pisa, 56126 Pisa, Italy; 5 Dipartimento Cardio Toraco Vascolare, Azienda Ospedaliero-Universitaria Pisana, 56126 Pisa, Italy; marielladesantis@libero.it; 6 Department of Cardiac, Thoracic, Vascular Sciences and Public Health, University of Padova, 35122 Padova, Italy; erica.bazzan@unipd.it; 7 Dipartimento di Oncologia, Azienda Ospedaliero-Universitaria Pisana, 56124 Pisa, Italy; andrea.sbrana@ao-pisa.toscana.it

**Keywords:** cancer, tissue factor, thromboembolism, extracellular vesicles

## Abstract

Cancer is characterized by chronic inflammation and hypercoagulability, with an excess of venous thromboembolism (VTE). Tissue factor, the initiator of blood coagulation, circulates associated with extracellular vesicles (EV-TF). Studies investigating EV-TF between cancer-associated and non-cancer-associated VTE are lacking. We therefore compared EV-TF in unprovoked VTE (U-VTE), cancer-associated VTE (C-VTE), and cancer without VTE (C-w/o VTE). We also investigated interleukin-6 (IL-6) levels between the same groups. The final population included 68 patients (U-VTE: *n* = 15; C-VTE: *n* = 24; C-w/o VTE: *n* = 29). All patients with VTE were enrolled within 48 h of diagnosis; non-VTE patients were recruited in the oncologic outpatient services. EV were isolated by differential centrifugation from 4 mL of peripheral blood; the final EV pellet (16,000× *g* for 45 min) was resuspended in 100 μL saline and tested for TF using a one-step clotting assay. There was a statistically significant difference for higher EV-TF in C-VTE and C-w/o VTE compared to U-VTE (*p* = 0.024; Kruskal–Wallis test). There was no significant difference between C-VTE and C-w/o VTE. Moreover, we did not find any difference in IL-6 levels. These preliminary data suggest that cancer represents, per se, a strong driver of EV-TF generation.

## 1. Introduction

Venous thromboembolism (VTE), including deep vein thrombosis and pulmonary embolism, represents the third leading cause of cardiovascular morbidity after coronary artery disease and stroke. Although VTE can present without an obvious underlying cause (the so-called unprovoked VTE), several well-defined risk factors for VTE have been described; among them, active cancer is one of most relevant [1]. Cancer-associated thromboembolism (CAT) is a major clinical concern, and the incidence of CAT has been progressively growing over the last decades. This increase is due to the increased survival of cancer patients and to the potential prothrombotic activity of anticancer therapies. An apparent increase in the incidence of CAT is also attributed to the widespread use of contrast-enhanced computed tomographic (CT) scans performed for oncologic follow-up, leading to the identification of asymptomatic or paucisymptomatic events [2]. Although CAT is associated with a worse prognosis in cancer patients, accepted prognostic biomarkers are still lacking, thus representing an unmet need in this area [3].

**Figure 1:**
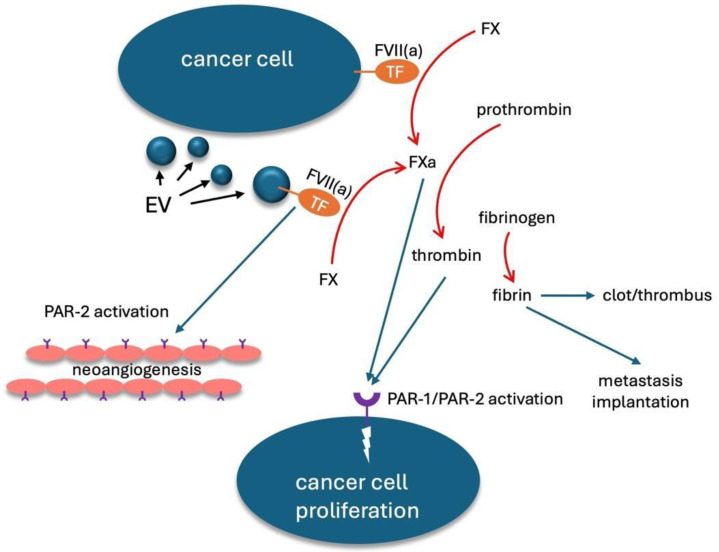
Schematic representation of tissue factor role in cancer. TF: tissue factor; FVII: coagulation factor VII; FVIIa: active FVII; FX: coagulation factor X; FXa: active FX; EV: extracellular vesicles. PAR: protease-activated receptor.

Extracellular vesicles (EV) are submicron vesicles produced by all eukaryotic cells, both constitutively and upon different specific stimuli (such as apoptosis, inflammation or acute infection). Their role in numerous diseases, including cardiovascular diseases and cancer, has been recognized [4,5]. EV can carry active molecules on their outer membrane or within their cytoplasm, and they can therefore have a role in biologic and pathologic processes [6]. Among these molecules, tissue factor (TF), a 46-KDa integral membrane protein which represents the physiological initiator of the coagulation cascade in vivo, plays a pivotal role in CAT. Beyond its role in the initiation of coagulation, in murine models TF has promoted tumor progression through interaction with protease-activated receptors (PARs), which are present on target cells like endothelial cells, platelets, or tumor cells themselves. This interaction induced relevant activities in the target cells, like the promotion of tumor cell survival, neoplastic angiogenesis, and, therefore, the development of distant metastasis [7]. A schematic representation of these complex mechanisms is depicted in Figure 1. EV-associated TF has also been studied as a potential prognostic biomarker in patients with CAT with some promising, even though not always concordant, results [8]. From a mechanistic point of view, thrombosis and inflammation are closely linked; indeed, the term “thromboinflammation” is currently used to depict this biologic interplay. Thromboinflammation is thought to be involved in many pathologic conditions, such as sepsis, cardiovascular and respiratory diseases, and cancer [9,10]. Interleukin (IL)-6 is a 26-KDa protein, and it represents one of the most important acute phase cytokines; its levels typically rise in different pathologic conditions, including thrombosis [11]. Interestingly, IL-6 is also involved in the pathogenesis of cancer through the promotion of the proliferation and survival of cancer cells as well as neoangiogenesis [12].

In this scenario, it is conceivable that thromboinflammatory processes involving procoagulant EV and inflammatory cytokines may be relevant in CAT. However, there are currently few non-conclusive data about the comparison of procoagulant EV in patients with acute VTE, with or without active cancer. We therefore investigated whether procoagulant EV and IL-6, as expression of thromboinflammation during VTE, would differ between patients with acute VTE, with or without active cancer, and cancer patients without VTE. We hypothesized that patients with VTE and active cancer would show the highest values of both EV-TF and IL-6 because of the concurrent presence of these two potential procoagulant and proinflammatory conditions, such as active cancer and acute thrombosis.

## 2. Materials and Methods

### 2.1. Subject and Study Design

We conducted a cross-sectional observational trial, and we enrolled three groups of subjects: (1) patients with acute unprovoked VTE (U-VTE), which means VTE without any obvious causal factor, including active cancer; (2) patients with acute, cancer-associated VTE (C-VTE); and (3) patients with active cancer but without VTE (C-w/o VTE).

Patients with both U-VTE or C-VTE were enrolled within 48 h of VTE diagnosis; C-w/o VTE patients were enrolled both in outpatient oncology clinics and in inpatient settings.

The main exclusion criteria were: age < 18 years, superficial vein thrombosis, uncertain VTE diagnosis (indeterminate computed tomographic pulmonary angiography (CTPA) or lung perfusion scan), presence of a major transient risk factor for VTE (for example recent surgery or immobilization), severe renal failure (estimated glomerular filtration rate < 15 mL/min/1.73 m^2^), concomitant infection or sepsis/septic shock, and concomitant acute arterial thrombosis (like acute coronary syndrome or stroke).

The main anthropometric and clinical data were recorded at enrollment; routine blood analysis was performed as per routine clinical practice. Each patient underwent the collection of venous blood samples for EV-associated TF activity (EV-TF), total plasmatic TF, and IL-6 measurement at enrollment.

This study was approved by the Ethic Committee of Area Vasta Toscana Nord-Ovest (protocol number 23835) and was conducted according to the principles of the Helsinki Declaration. Each participant signed a written informed consent form.

### 2.2. Diagnosis of VTE

Acute VTE was diagnosed as per clinical practice. In particular, unprovoked pulmonary embolism was diagnosed with computed tomographic pulmonary angiography (CTPA; Siemens X cite CT scan) or lung perfusion scan, according to current guidelines [1]. Cancer-associated pulmonary embolism was detected alternatively during a contrast-enhanced CT scan performed for routine oncologic follow-up, or it was diagnosed according to current guidelines when there was clinical suspicion, like for U-VTE [1]. Deep vein thrombosis was diagnosed with Doppler ultrasonography or vein CT scan, according to the clinical presentation.

### 2.3. EV Isolation from Patients Blood and Analysis of EV-TF, Plasmatic TF, and Cytokine Concentration

The isolation of EVs was identical to that previously published [13]. Briefly, a sample of 8 mL peripheral blood was withdrawn into sodium citrate (0.9% *w*/*v*) and immediately submitted to low-speed centrifugation (1500× *g* for 15 min at 4 °C) to remove whole cells and cell debris. After that, platelets were separated to obtain platelet-poor plasma (PPP) using high-speed centrifugation (16,000× *g* for 2 min at 4 °C). A portion of the PPP was frozen for later analysis of circulating IL-6 and TF. Medium-large EVs were finally sedimented by a further high-speed centrifugation step (16,000× *g* for 45 min at 4 °C). The pellet was resuspended in 100 µL normal saline and stored at −80 °C until analysis, with the resuspended pellet used to evaluate EV-associated TF.

To test EV-TF, a one-stage clotting assay was performed using a semi-automated coagulation analyzer (Start Max, Diagnostica Stago, Milan, Italy), as described in [14]. Briefly, the test was based on the clotting time upon recalcification of citrated normal plasma with 25 mM CaCl_2_; TF availability was the rate-limiting factor for this reaction. For each experimental session, calibration curves were generated using recombinant human relipidated TF (pg/mL) (BioMedica Diagnostics, Windsor, NS, Canada). Under our experimental conditions, clotting times of 19 ± 2 s and 627 ± 84 s were obtained with 100 pg/mL and 0.001 pg/mL TF, respectively.

Plasma concentrations of IL-6 (Sinobiological, Wayne, PA, USA) and TF (Abcam, Cambridge, UK) were measured by a sandwich ELISA kit with a microplate reader (iMarkTM Microplate Absorbance Reader, Bio-Rad, Milan, Italy) according to the manufacturer’s instructions.

### 2.4. Statistical Analysis

We used the Shapiro–Wilk test to assess the data distribution; normally distributed variables were then reported as means ± standard deviations, while non-normally distributed variables were reported as medians [interquartile range]. The Kruskal–Wallis test or one-way ANOVA were used for multiple comparisons among independent groups, as appropriate; the Fisher exact test was used to compare categorical variables. A *p* < 0.05 was chosen to assess statistical significance.

Jamovi software (version 2.3.21.0 for MacOS, available at www.jamovi.org, accessed 5 December 2022) was used for statistical analysis; Prism software (v. 9.4.1 for MacOS; GraphPad, San Diego, CA, USA) was used for figure preparation.

## 3. Results

### 3.1. Study Population

The enrolled population included 71 subjects, but we decided to exclude from the final analysis two patients (one belonging to U-VTE group and one belonging to C-VTE group) because their EV-TF values were more than five-fold the median value of their groups and one patient belonging to the C-w/o VTE group whose IL-6 values were almost 30-fold the median value. The laboratory results were confirmed on repeated tests. We therefore considered these three subjects as real outliers. Thus, the final population (on which we performed all the following analysis) included 68 patients, divided into the above-mentioned three groups as follows: 15 patients in the U-VTE group, 24 patients in the C-VTE group, and 29 patients in the C-w/o VTE group.

We report the main anthropometric and baseline laboratory data of the study subjects in Table 1. The three groups were homogeneous except for hemoglobin and hematocrit values, which were significantly lower in patients with active cancer, irrespective of the presence of VTE, than in patients with U-VTE.

In Table 2, we reported the site of the primary cancer in oncologic patients (C-VTE and C-w/o VTE). There was a clear predominance of lung cancer in C-w/o VTE patients. Thirteen C-VTE patients (54% of total) presented with metastatic disease, while 17 C-w/o VTE patients (57% of total) presented with metastatic disease at enrolment.

### 3.2. Prothrombotic Activity

**Figure 2:**
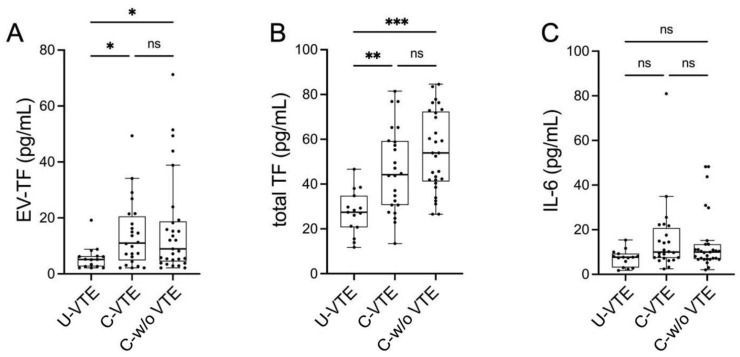
Evaluation of (**A**) EV-TF (pg/mL), (**B**) total TF (pg/mL), and (**C**) IL-6 (pg/mL) in the three study groups: U-VTE, C-VTE and C-w/o VTE. * *p* < 0.05; ** *p* < 0.005; *** *p* < 0.001; ns: not significant. EV-TF: extracellular vesicles-associated tissue factor activity; TF: tissue factor; IL-6: interleukin-6; U-VTE: unprovoked venous thromboembolism; C-VTE: venous thromboembolism with active cancer; C-w/o VTE: active cancer without venous thromboembolism.

We first analyzed EV-TF, and we found that the distribution showed a significant difference among the three groups (*p* = 0.024 with Kruskal–Wallis test). In the post hoc analysis performed with the Dwass–Steel–Critchlow–Flinger test, there was a statistically significant difference between U-VTE and C-VTE (*p* = 0.031) and between U-VTE and C-w/o VTE (*p* = 0.044). There was no difference between C-VTE and C-w/o VTE (*p* = 0.998) (Figure 2A). We then compared total plasmatic TF concentration; similar to EV-TF, the distribution showed a significant difference among the groups: again, in the post hoc analysis, there was a statistically significant difference between U-VTE and C-VTE (*p* = 0.005) and between U-VTE and C-w/o VTE (*p* < 0.001) (Figure 2B).

**Figure 3:**
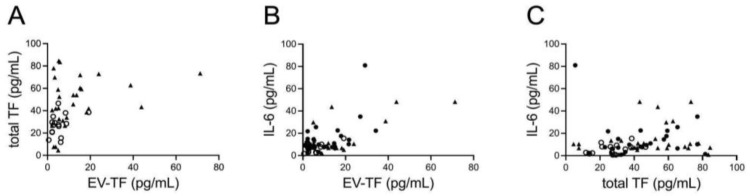
Direct correlation in the whole study population. (**A**) EV-TF and total TF (Spearman rho 0.491, *p* < 0.001), (**B**) IL-6 and EV-TF (Spearman rho 0.380, *p* = 0.001) and (**C**) IL-6 and total TF (Spearman rho 0.405, *p* < 0.001). U-VTE = empty circles; C-w/o VTE = triangles; C-VTE = filled circles; EV-TF: extracellular vesicles-associated tissue factor activity; TF: tissue factor; IL-6: interleukin-6; U-VTE: unprovoked venous thromboembolism; C-VTE: venous thromboembolism with active cancer; C-w/o VTE: active cancer without venous thromboembolism.

There was a moderate direct correlation between EV-TF and plasmatic TF in the whole study population (Spearman rho 0.491, *p* < 0.001) (Figure 3A). When analyzing the three groups separately, we found a strong and significant correlation between EV-TF and plasmatic TF only in the C-VTE group (Spearman rho 0.795, *p* < 0.001).

### 3.3. Circulating IL-6 Levels

The distribution of circulating IL-6 levels did not show statistically significant differences among the three groups (*p* = 0.077 with Kruskal–Wallis test) (Figure 2C). While we found a weak-to-moderate direct correlation between IL-6 and both EV-TF and plasmatic TF in the whole population (Spearman rho 0.380, *p* = 0.001 and Spearman rho 0.405, *p* < 0.001, respectively) (Figure 3B,C), when we analyzed the three groups separately, we found a significant correlation only in C-VTE (Spearman rho 0.501, *p* = 0.013 with EV-EF-TV and Spearman rho 0.533, *p* = 0.007 with plasmatic TF).

## 4. Discussion

In this study, we found that patients with active cancer have higher levels of EV-TF and total plasmatic TF, irrespective of the presence of associated VTE, compared to patients with U-VTE. No significant differences were found in IL-6 levels among the three groups.

Previous studies have evaluated the hypothesis that EV-TF levels are higher in patients with CAT. Tesselaar et al. conducted a study with a design and results similar to ours: indeed, they found that EV-TF was higher in patients with active cancer with or without VTE than in U-VTE, but no difference emerged between the two groups with active cancer (C-VTE and C-w/o VTE). However, there were few patients with cancer with VTE, and there was significant heterogeneity regarding the cancer stage (local versus metastatic disease) [15]. The same group, therefore, designed a new study including only patients with cancer with or without VTE. Patients were quite homogeneous regarding tumor histotype and stage. The authors found that C-VTE had higher EV-TF than C-w/o VTE patients [16]. Similar results were obtained in another study investigating only patients with pancreatic cancer, in which EV-TF was significantly higher in patients with C-VTE than in the C-w/o VTE group, even though C-VTE patients represented a small minority of total cancer patients (ratio between patients with and without VTE approximately 1:6) [17]. Last, a study by Hisada et al. compared EV-TF in patients with active cancer and severe acute non-neoplastic illness (e.g., infection, stroke, VTE, exacerbation of chronic obstructive pulmonary disease) and found significantly higher EV-TF values in cancer patients. Interestingly, patients with adenocarcinoma had higher EV-TF than non-adenocarcinoma histotype; however, no difference was found in EV-TF between patients with adenocarcinoma and VTE and patients with adenocarcinoma but without VTE, even though the sample size was quite small [18]. In all these studies, EV-TF was evaluated in terms of procoagulant activity, using a clotting assay that measured factor Xa generation depending on TF availability; although we took a slightly different approach, we too analyzed procoagulant activity. It is conceivable that the heterogeneity in some of the results depends on the different sample sizes and the inhomogeneity in the study populations. Other cross-sectional studies investigated EV-TF using a different approach. These studies did not evaluate the EV-associated procoagulant activity, but rather enumerated EV-TF antigen by flow cytometry. Two studies found higher levels of EV-TF in C-VTE than in both C-w/o VTE and U-VTE [19,20]. Nevertheless, the different methodological approaches (functional versus antigenic) strongly limit the possibility of comparing the data from these experiments. It is known that TF activity partly depends on the reversible form that TF can assume, a process called “encryption and decryption”. The encrypted TF has a weak procoagulant activity, and the decryption process, which is associated with the active form of TF, is likely related, at least in part, to the availability of phosphatidylserine (PS) in the cellular or EV outer membrane [21]. Interestingly, while PS is usually abundant on EV membranes, a small population of medium-to-large, PS-negative EV has been described [22]. In this scenario, we can speculate that EV-TF measured through flow cytometry might also include PS-negative EV, which cannot promote TF decryption, thus resulting as inactive in the coagulation process. Of course, these EV-TF would not be detected in a functional assay like the one we used in this study. Taken together, however, these data are in line with ours, suggesting that the presence of active cancer seems to represent the main driver of EV-TF production and activity. We actually found only one study that reported higher EV-TF levels, measured through flow cytometry, in U-VTE than in C-VTE [23]; to our knowledge, similar data have not been replicated in other studies.

The vast majority of the studies published on TF and CAT investigated EV-associated TF, and we did not find any cross-sectional studies on this topic. Nevertheless, there are some prospective studies aiming to identify TF as a biomarker of CAT. In a study conducted on patients with pancreatic cancer, significantly higher levels of total circulating TF (evaluated through an ELISA kit) were found at baseline in patients who further developed CAT during the study follow-up than in patients who did not. Interestingly, the authors found a significant correlation between total circulating TF and EV-TF activity only in patients with CAT, as we showed in our study [24]. Noteworthy, a previous prospective study on patients with pancreatic cancer did find a significant correlation between circulating TF and EV-TF activity in both the whole study population (all patients with cancer, with or without VTE) and in the subgroup of CAT. In that study, however, the very small sample size (the total population included 11 patients, with only 2 patients who developed CAT during the follow-up period) probably limited the strength of the conclusions [25]. Again, a possible explanation for these conflicting data can rely on the complex “encryption and decryption” process that can theoretically be different in patients with cancer with or without VTE. Actually, TF has pleiotropic roles in cancer, which go far beyond blood coagulation: indeed, TF is involved in cell survival, tumor angiogenesis and distant metastasis production [7]. It is, thus, conceivable that the differences that we and others observed between circulating TF and EV-TF activity may be partly due to these different activities of TF in cancer. Last, it is remarkable that different commercial ELISA kits for TF measurement can lead to different measurements of the total protein content, probably because of different sites of glycosylation on TF, which can alter the interaction with anti-TF antibodies [24].

We had also hypothesized a significant difference in IL-6 levels among our three groups of patients; however, the results are in clear contrast with our hypothesis. We did, however, observe a statistically significant correlation between IL-6 levels and EV-TF and TF. When the three groups were analyzed separately, this correlation was maintained only in CAT; in contrast, no statistically significant correlation was observed between IL-6 and EV-TF in C-w/o VTE or in U-VTE. We cannot explain these potentially intriguing results, which could represent a statistical artifact. Further prospective studies will investigate these correlations as prespecified analyses, thus providing new insights into this interesting interplay between inflammation and thrombosis. Actually, a possible mechanistic link between IL-6 and thrombosis in cancer has been hypothesized in a murine model [26], but, to the best of our knowledge, there are no studies investigating the relationships between IL-6 and both total and EV-associated TF in patients with cancer. Some previous studies have shown higher circulating levels of IL-6 in patients with deep vein thrombosis than in healthy controls [27,28]. However, the presence of active cancer was a clearly specified exclusion criterion in these studies, thus limiting the comparison with our data.

Our study has some limitations. First, the relatively small sample size did not allow for definitive conclusions, even though the aforementioned studies were conducted on similar samples. Most importantly, our C-w/o VTE group was not homogenous, since approximately 70% of patents had lung cancer, mainly with metastatic disease, thus limiting the applicability of our data to other types of neoplasms. Third, we did not investigate the cell origin of the EV: In particular, while, in U-VTE, circulating cancer-derived EV should be visually absent (with the possible exception of occult cancers not detectable at the time of VTE diagnosis), in C-VTE and C-w/o VTE, they are present by definition, but we do not know in which proportion they contribute to EV-TF activity. Fourth, since we focused on venous thromboembolism, we decided to exclude patients with acute arterial thrombosis such as ischemic stroke or myocardial infarction, even though the pivotal role of TF and IL-6 in atherothrombosis is well known, and the comparison with such a group could therefore be interesting [29]. Finally, the cross-sectional design did not allow us to identify EV-TF as reliable biomarkers of VTE risk development in cancer patients: some studies have already tried to investigate this topic, but with conflicting results so far [30]. A prospective study aimed at investigating the role of EC-TF in predicting VTE in lung cancer patients is currently underway.

## 5. Conclusions

In conclusion, our study showed significantly higher EV-associated prothrombotic activity in patients with active cancer, irrespective of the presence of active VTE, than in patients with U-VTE. We also found a relationship between EV-TF and IL-6, a marker of systemic inflammation also involved in cancer progression and in VTE, only in patients with C-VTE. These data suggest that cancer represents, per se, the main driver of EV-bearing TF production. It is, therefore, conceivable that EV-TF exert relevant biologic activities (beyond blood coagulation) in cancer, probably in a thromboinflammatory milieu, and this possibility deserves further mechanistic studies.

## Figures and Tables

**Table 1:** Baseline characteristics of the study population (*n* = 68). BMI: body mass index; WBCs: white blood cells; aPTT: activated partial thromboplastin time; INR: international normalized ratio; eGFR: estimated glomerular filtration rate. NS: not significant. Data are presented as medians (interquartile range) or means ± standard deviation, as appropriate.

	U-VTE (*n* = 15)	C-VTE (*n* = 24)	C-w/o VTE (*n* = 29)	*p*
Sex (M/F)	9/6	11/13	16/13	NS
Age, years [missing values]	70.5 ± 13.8 [0]	66.9 ± 12.0 [0]	69.6 ± 9.6 [0]	NS
BMI, kg/m^2^ [missing values]	27.7 ± 3.8 [5]	24.6 ± 4.6 [3]	24.7 ± 4.8 [0]	NS
Total WBC, mL^−1^ [missing values]	8010 (2640) [0]	7745 (6150) [0]	9520 (5140) [0]	NS
Neutrophils, mL^−1^ [missing values]	5594 (2843) [0]	5519 (5304) [0]	6120 (4170) [0]	NS
Lymphocytes, mL^−1^ [missing values]	1800 (1154) [0]	1309 (777) [0]	1320 (1010) [0]	NS
Hemoglobin, g/dL [missing values]	14.8 (2.1) [0]	11.3 (2.2) [0]	11.6 (1.4) [0]	<0.001 U-VTE vs. C-VTE <0.001 U-VTE vs. C-w/o VTE
Hematocrit, % [missing values]	42.0 (5.3)	33.7 (6.8)	35.3 (4.6)	<0.001 U-VTE vs. C-VTE <0.001 U-VTE vs. C-w/o VTE
Platelets, ×10^3^/mL [missing values]	194 (65) [0]	218 (100) [0]	265 (125) [0]	NS
aPTT, s [missing values]	29.4 (3.2) [1]	28.1 (3.9) [2]	27.6 (5.5) [2]	NS
INR [missing values]	1.02 (0.08) [1]	1.12 (0.20) [1]	1.07 (0.20) [2]	NS
Creatinine, mg/dL [missing values]	0.98 ± 0.24 [0]	0.88 ± 0.31 [0]	0.86 ± 0.31 [0]	NS
eGFR, mL/min/1.73 m^2^ [missing values]	84.6 ± 29.8 [2]	81.0 ± 29.5 [2]	81.9 ± 35.6 [0]	NS

**Table 2:** Site of primary cancer in oncologic patients.

Site of Primary Cancer	C-VTE (*n* = 24)	C-w/o VTE (*n* = 29)
Blood, *n*	2	0
Prostate, *n*	0	1
Lung, *n*	9	20
Kidney, *n*	5	0
Pancreas, *n*	3	2
Colon, *n*	2	1
Ovary, *n*	1	0
Melanoma, *n*	1	0
Breast, *n*	1	2
Liver and biliary tract, *n*	0	2
Cardias, *n*	0	1

## Data Availability

The raw data supporting the conclusions of this article will be made available by the authors on request.
